# Fermented Feed Modulates Meat Quality and Promotes the Growth of *Longissimus Thoracis* of Late-Finishing Pigs

**DOI:** 10.3390/ani10091682

**Published:** 2020-09-17

**Authors:** Yueqin Qiu, Kebiao Li, Xichen Zhao, Shilong Liu, Li Wang, Xuefen Yang, Zongyong Jiang

**Affiliations:** 1 Institute of Animal Science, Guangdong Academy of Agricultural Sciences, Guangzhou 510640, China; qiuyueqin87@126.com (Y.Q.); likebiao1996@163.com (K.L.); liushilong94@126.com (S.L.); wangli1@gdaas.cn (L.W.); jiangz38@gmail.com (Z.J.); 2 State Key Laboratory of Livestock and Poultry Breeding, Guangzhou 510640, China; 3 Key Laboratory of Animal Nutrition and Feed Science in South China, Ministry of Agriculture and Rural Affairs, Guangzhou 510640, China; 4 Guangdong Provincial Key Laboratory of Animal Breeding and Nutrition, Guangzhou 510640, China; 5 Maoming Branch, Guangdong Laboratory for Lingnan Modern Agriculture, Guangzhou 510640, China; 6 College of Animal Science, South China Agricultural University, Guangzhou 510642, China; xichzhao@163.com

**Keywords:** fermented diet, meat quality, mTOR, proteomic analysis, muscle growth, finishing pigs

## Abstract

**Simple Summary:**

Little is known about the effect of fermented feed on lean mass production of finishing pigs and the underlying mechanism. This study aimed to investigate the effect of fermented diet on growth performance, carcass traits, meat quality and growth of longissimus thoracis (LT) of finishing pigs. Our results found that fermented diet significantly increased loin eye area and lean mass percentage, decreased backfat thickness and improved meat quality of LT by decreasing shear force and drip loss at 48 h post slaughter and improving meat sensory characteristics compared with control diet. Fermented diet also increased the abundance of insulin, insulin receptor, myoblast determination protein (*MyoD*) and myosin heavy chain-I (*MyHC-I*) transcripts, and the phosphorylation levels of protein kinase B (AKT), mammalian target of rapamycin (mTORC1), initiation factor 4E-binding protein 1 (4EBP1) and S6 kinase 1 (S6K1) in LT, while decreasing the expression of muscle atrophy F-box (*MAFbx*) and forkhead Box O1 (*Foxo1*) mRNA transcripts. Our results indicated that fermented diet improved meat quality and enhanced LT growth of finishing pigs by increasing insulin/AKT/mTORC1 protein synthesis cascade and activating Foxo1/MAFbx pathway, along with the regulation of ribosomal protein and proteins involved in muscle contraction and muscle hypertrophy.

**Abstract:**

This study investigated the effect of fermented diet on growth performance, carcass traits, meat quality and growth of longissimus thoracis (LT) of finishing pigs. A total of 48 finishing pigs [Duroc × (Landrace × Large White), male, 126 ± 5-d-old] weighing 98.76 ± 1.27 kg were randomly assigned to two treatments (eight pens per treatment and three pigs per pen) for a 28-d feeding trial, including control diet and fermented diet. Fermented diet significantly increased the loin eye area and lean mass percentage, decreased backfat thickness and improved meat quality of LT by decreasing the shear force and drip loss at 48 h post slaughter and improving meat sensory characteristics compared with control diet. A fermented diet also significantly increased the abundance of insulin, insulin receptor (*IR*), myoblast determination protein (*MyoD)* and myosin heavy chain-I (*MyHC-I*) transcripts, and the phosphorylation levels of AKT, mTORC1, 4EBP1 and S6K1 in LT, while decreasing the expression of muscle atrophy F-box (*MAFbx*) and forkhead Box O1 (*Foxo1*) mRNA transcripts. Moreover, proteomic analysis revealed that differentially expressed proteins predominantly involved in protein synthesis and muscle development were modulated by fermented diet. Our results indicated that a fermented diet improved meat quality and enhanced LT growth of finishing pigs by increasing insulin/AKT/mTORC1 protein synthesis cascade and activating the Foxo1/MAFbx pathway, along with the regulation of ribosomal protein and proteins involved in muscle contraction and muscle hypertrophy.

## 1. Introduction

Fermented feed, which was fermentation of plant materials with beneficial bacterial, has been extensively studied in attempts to improve feed utilization and decrease use of in-feed antibiotics [1]. Microbial fermentation could degrade anti-nutritional factors in feed and improve nutritional value [2,3,4]. Zheng et al. observed an increase in crude protein content and a decrease in glycinin and β-conglycinin during Bacillus siamensis fermentation of soybean meal [5]. Shi et al. found that fermentation of rapeseed meal with Aspergillus niger increased small-sized peptides and amino acid profiles while reducing levels of neutral detergent fiber (NDF), glucosinolates, isothiocyanate and phytic acid. Fermented feed is enriched in beneficial microbiota and bacterial metabolites, which may contribute to improving the gastrointestinal microbial ecosystems of pigs [6,7,8,9,10]. Additionally, fermentation using lactic acid-producing microbes leads to a decrease in pH value of the substrates, and thus, presents beneficial effects to both the feed during storage and the animals consuming fermented products [11,12]. 

The effect of fermented feed on growth performance, gut microbiota and immunity of piglets has been widely reported [3,13]. However, only a few studies evaluated the effect of fermented feeds on the growth performance and meat quality of finishing pigs with inconsistent results. Lee et al. found that fermented apple pomace diet supplementation improved ADG (average daily gain) and feed efficiency in finishing pigs [14]. The results from Xu et al. also showed that ADFI (average daily feed intake) and ADG were greater in finishing pigs fed fermented feed than those fed control diet [15]. However, Liu et al. observed that dietary corn bran fermented by B. subtilis did not affect the ADFI and ADG of finishing pigs [16]. Hu et al. reported that there was no difference in growth performance between finishing pigs fed solid-stated fermented feed and basal diet [17]. Thus, the effects of fermented feed on growth performance needs further exploration.

Several studies have investigated the effect of fermented feed on meat quality of finishing pigs and focused on lightness, redness, shear force, flavor and drip loss [14,18,19]. However, few studies reported the effect of fermented feed on skeletal muscle mass, except the study by Ahmed et al., which showed that fermented herb diet increased lean production [20], but the mechanisms underlying this observation remains unknown. To our knowledge, increased muscle mass is one of the major objectives of animal production, highlighting the need to elucidate the molecular mechanisms underlying fermented feed regulation of lean production [21]. 

Thus, the present study aimed to investigate the effect of fermented feed on growth performance, meat quality and muscle growth in pig during the late-finishing phase. The underlying mechanism of skeletal muscle growth of LT modulated by fermented feed was also explored.

## 2. Materials and Methods 

All animal procedures used in this study were approved by the Animal Care and Use Committee of Guangdong Academy of Agricultural Sciences (authorization number GAASIAS-2016-017). All efforts were made to minimize any suffering of animals in accordance with the Chinese Guidelines for Animal Welfare of Science and Technology Ministry of China (C/T 35892–2018, Beijing, China).

### 2.1. Diets Preparation and Laboratory Analyses

The basal diet (control diet) was formulated to meet the nutrient recommendations of the National Research Council 2012 (NRC, 2012) (Table 1). Both control and fermented diets were prepared on site 1 week before the experiment. Fermented diet was prepared using basal diet followed by solid-state fermentation, which was a fermented technique involving microbiota growing on solid material under controlled conditions in the absence of free water [22]. In total, 1000 kg substrates was evenly mixed with 420 L microbial suspensions consisting of Saccharomyces cerevisiae (1.5 × 10^8^ cfu/mL), Bacillus subtilis (2 × 10^7^ cfu/mL) and Lactobacillus reuteri (1.8 × 10^8^ cfu/mL). The mixture was then transferred to a polythene bag (20 kg in capacity) equipped with a one-way valve at room temperature (25–35 °C) for at least 96 h. After fermentation, wet fermented samples (approximately 800 g) were collected for chemical analysis; the remaining fermented feed with a moisture content of 38 ± 2.5% was kept in a room at ambient temperature (16 °C). 

Both the fermented sample and basal diet (3 g) were suspended in 10 mL ultrapure water and centrifuged at 5000× *g* for 15 min. The supernatant was collected for pH and lactic acid analysis. pH was measured using an electronic pH meter (HI 8242C, Beijing Hanna Instruments Science & Technology, Beijing, China). The lactic acid content was determined using a lactic acid assay kit in accordance with the protocol of the manufacturer (Nanjing Jiancheng Institute of Bioengineering and Technology Nanjing, China). For chemical composition analysis, samples of control and fermented feeds were dried at 65 °C for 24 h and then ground using a hammer mill and sifted through a 1 mm screen for dry matter, crude fiber, neutral detergent fiber, acid detergent fiber, crude fat, starch, crude protein contents and amino acids analysis. Crude fiber, neutral detergent fiber, acid detergent fiber, crude fat, starch and crude protein contents of the sample were measured by the methods of the Association of Official Analytical Chemists (AOAC, 2007) and calculated on an 88% dry matter basis. The amino acid profiles of feed samples were determined using an AA analyzer (Hitachi L8800, Tokyo, Japan) by the post-column derivatization of ninhydrin. Before analysis, 0.1 g sample was hydrolysis with 10 mL 6 M HCl for 24 h at 115 °C. Hydrolyzates were then transferred to a 50 mL volumetric flask and diluted with 0.02 mol/L HCl. 1 mL diluent was transferred to a 6 cm glass dish and evaporated to dryness in a water bath at 65 °C. After dissolving with 2 mL HCl (0.02 mol/L) and filtering (0.22 μm), amino acids were fractionated on a protein hydrolysate analysis column (HISCO-855-4506, Hitachi) at 0.40 mL/min outflow rate. The individual amino acid was identified using standards (013-08391, Wako, Tokyo, Japan) and calculated on an 88% dry matter basis. 

### 2.2. Animals and Experimental Design

A total of 48 finishing pigs [Duroc × (Landrace × Large White), male, 126 ± 5-d-old] weighing 98.76 ± 1.27 kg were randomly assigned to two treatments (eight pens per treatment and three pigs per pen). The pigs were provided with a control diet or fermented diet entirely substitute for control diet. All pigs were housed in 16 adjacent pens (1.8 × 4.6 m) equipped with slatted floors in an environmentally-controlled facility. The pigs were fed three times a day (at 7:30 am, 12:30 am and 18:30 pm) with the prepared diet in feeding troughs and had ad libitum access to feed and water. Feed leftovers in each pen were collected and quantified every day. The amount of feed consumed per pen was recorded every day to determine the average daily feed intake (ADFI) on an 88% dry matter basis. All pigs were weighed individually at the beginning and the termination of the experiment to calculate the average daily gain (ADG). The feed to gain ratio (F/G) were calculated from the feed intake (on an 88% dry matter basis) and the increased body weight during the whole experimental period.

### 2.3. Carcass Measurement and Tissue Sampling

At the end of 39 d treatment (by when the body weight (BW) of control pigs were about 130 kg, 165 ± 5-d-old), one pig from each pen with medium BW in both control and experimental group was selected and transferred to the slaughterhouse within the Institute of Animal Science, Guangdong Academy of Agricultural Sciences. The pigs were electrically stunned, exsanguinated, eviscerated and divided lengthwise. The backfat depths over the first, 10th and last ribs were measured using a vernier caliper (Guangzhou Tool Factory, Guangzhou, China). Loin eye area was traced at the interface of 10th to 11th rib and measured by planimetry. Percentage of lean mass was estimated according to the guidelines of National Research Council (NRC) 1998: lean mass (%) = 4.40 + 0.54 × carcass weight (kg) − 4.21 × average back fat thickness (cm) + 0.18 × loin eye area.

### 2.4. Meat Quality Measurements

LT muscle samples (on the right side of the carcass, at the level of the 10th to 14th ribs) were removed for meat quality traits analysis. A chop (25 mm thick slice) for meat color and pH measurements was excised from LT sample between the 10th and 11th ribs. Meat color values (lightness, L *; redness, a *; and yellowness, b *) were measured at 45 min, 24 h and 48 h after slaughter using a Minolta CR-400 Chroma meter (Konica Minolta Sensing Ins., Osaka, Japan) with an 8 mm measuring port, D65 illuminant and a 10° observer angle, and the values of L *, a * and b * of each sample were recorded in triplicate. The pH of LT samples at 45 min, 24 h and 48 h post-mortem were determined using a pH meter (HI 8242C, Beijing Hanna Instruments Science & Technology, Beijing, China), which was calibrated with pH 4.6 and 7.0 buffers before measurements.

A chop (25 mm thick slice) was excised for shear force (N) analysis from the LT sample between the 11th and 12th ribs, vacuum packed and stored at 4 °C. At 24 h after slaughter, the muscle samples were placed in a plastic bag and cooked in a 80 °C water bath until the internal temperature reached 70 °C. Internal temperature was monitored during cooking with a hand-held temperature probe placed into the geometric center of each sample. Then cooked samples were cooled in running water to room temperature and were sheared parallel to the muscle fiber direction and cut into shaped strips (1 cm × 1 cm × 3 cm). Finally, the shear force (N) of the LT was measured using a digital-display-muscle tenderness meter (C-LT3B, Tenovo, Harbin, China) with a load cell of 15 kg and a 200 mm/min crosshead speed. Fifteen replicates of each sample were recorded. 

A chop (30 mm thick slice) was excised for drip loss analysis from the LT sample between the 13th and 14th ribs. Drip loss of LT samples were determined on weighed cubes of muscle (about 30 g), suspended on hooks in airtight plastic bags, stored at 4 °C for 24 h and 48 h and reweighed after removing surface moisture. Drip loss was calculated as:

[(initial weight − final weight)/initial weight] × 100.(1)

Intramuscular fat content was determined using Soxhlet extraction [23]. Approximately 30 g of fresh LT sample from the 10th and 11th ribs was processed in a meat grinder (Philips, Eindhoven, The Netherlands); then, we removed the visible fascia, which was lyophilized using freeze-drying system (Marin Chris, Osterode, Germany) and then ground. The powder (3 g) was extracted with petroleum ether (30 to 60 °C boiling point) and analyzed for lipids using the Soxtec 2055 fat extraction system (Foss Tecator AB, Hilleroed, Denmark), according to the procedures of AOAC 2007. 

A chop (25 mm thick slice) that was excised for sensory evaluation from the LT sample between the 12th and 13th ribs was vacuum packed and stored in a refrigerator (4 °C). At 4 h after slaughter, meat samples were taken out, trimmed and cut into eight cubes of about 1.8 cm dimension. Each sample cube was placed in a bowl, sealed with aluminum foil and labeled with a random code. Bowls containing samples were cooked at 120 °C in a humid oven for 10 min then cooled in a water bath (50 °C) until served to eight panelists, who have experience in sensory assessment, across one session. There was about 4 min for panelists to evaluate each sample and each panelist was asked to test 16 samples for seven subjective traits, including tenderness, juiciness, flavor, fragrance, off-flavors, broth freshness and color. Distilled water (about 30 °C) was provided for panelists to cleanse their palate between samples. Sensory variables were rated using eight-point scales: tenderness (1 = very tough, 8 = very tender), juiciness (1 = very dry, 8 = very juicy), flavor (1 = very poor, 8 = very good), fragrance (1 = very weak, 8 = very strong), off-flavors (1 = very strong, 8 = very weak), broth freshness (1 = very poor, 8 = very strong) and color (1 = pale, 8 = dark purplish red).

### 2.5. Quantitative Real-Time PCR (qPCR)

LT sample between the 10th and 11th ribs was immediately collected and frozen in liquid nitrogen, and then stored at −80 °C for qPCR. Trizol reagent (Invitrogen, Carlsbad, CA, USA) was used to isolate total RNA from LT sample, according to the manufacturer’s instructions. Purity and concentrations of RNA were assessed using a NanoDrop-ND1000 spectrophotometer (Thermo Fisher Scientific Inc., Walldorf, Germany). Total RNA (1 μg) was used to synthesize cDNA using a PrimeScript™ II 1st Strand cDNA Synthesis Kit (Takara, Tokyo, Japan). SYBR green I (Takara, Dalian, China), 10-fold diluted cDNA and gene-specific primers (Table 2) in a final volume of 20 μL were used to perform qPCR analyses in triplicate. Conditions for qPCR were 95 °C × 3 min, then 40 cycles of amplification were performed (95 °C × 15 s, 60 °C × 30 s, 72 °C × 30 s). The β-actin transcript was used as an internal control because its abundance was not markedly influenced by treatment in the present study (results not shown). The mRNA abundance of the target genes, relative to β-actin was analyzed using the 2−ΔΔCt method, ΔCt = Ct (target gene) − Ct (β-actin) and ΔΔCt = ΔCt (Treatment) − ΔCt (Control). 

### 2.6. Western Blot Analysis

An LT sample between the 10th and 11th ribs was immediately collected and frozen in liquid nitrogen, and then stored at −80 °C for western blot analysis. About 0.5 g frozen sample was lysed for 30 min at 4 °C in 1 mL of RIPA buffer containing 1% protease inhibitor cocktail and 1% phosphatase inhibitor. The sample lysates were centrifuged (13,000× *g* for 15 min at 4 °C). Concentrations of extracted protein were measured by a BCA protein assay kit (Pierce, Rockford, IL, USA) with bovine serum albumin (BSA) standards. Equal quantities of protein were diluted with 5× loading buffer, denatured at 100 °C for 10 min, cooled on ice and then used for Western blots. Denatured proteins were separated by 10% sodium dodecyl sulfate polyacrylamide gel electrophoresis (SDS-PAGE), and then, separated proteins were transferred to nitrocellulose membranes. After blocking with 3% BSA in tris buffered saline tween (TBST) buffer for 10 min, membranes were washed three times, then incubated with the diluted primary antibodies overnight at 4 °C. Subsequently, after washing three times, membranes were then incubated with the appropriate HRP-labeled secondary antibodies for 1 h at room temperature. After three washes, immunoreactive proteins were visualized with chemiluminescent HRP substrate (Millipore, Billerica, MA, USA) and a VersaDoc imaging system (Bio-Rad, Hercules, CA, USA). Image J software (National Institutes of Health, Bethesda, MD, USA) was used to quantify the band of protein intensity. Primary antibodies for β-actin (Cat number: #4970), p-AKT (Cat number: #4060), p-mTOR (Cat number: #2971S), p-4EBP1 (Cat number: #62855S) and p-S6K1 (Cat number: #2215S) were all from Cell Signaling Technology, Boston, MA). The results are expressed as the abundance of each target protein relative to β-actin.

### 2.7. Proteome Analysis

Protein digestion of LT sample from the 10th and 11th rib was performed according to the filter aided sample preparation (FASP) procedure described by Wisniewski et al., and the resulting peptide mixture was labeled using the 4-plex/8-plex iTRAQ reagent according to the manufacturer’s recommendations (Applied Biosystems, Foster City, CA, USA) [24]. Briefly, 200 μg of proteins from each sample were added to 30 μL STD buffer (4% SDS, 100 mM DTT, 150 mM Tris-HCl pH 8.0), then the detergent dithiothreitol (DTT) and other low-molecular-weight components were removed using UA buffer (8 M Urea, 150 mM Tris-HCl pH 8.0) by multiple ultrafiltration (Microcon units, 30 kD). To block the cysteine residues, 100 μL 0.05 M iodoacetamide was added into UA buffer and the mixture were incubated for 20 min in the darkness. The filters were washed three times with 100 μL UA buffer and then twice with 100 μL DS buffer (50 mM triethylammoniumbicarbonate at pH 8.5). Finally, 2 μg trypsin (Promega, Madison, WI, USA) in 40 μL DS buffer was used to digest the protein suspensions overnight at 37 °C, and then the resulting peptides were collected as a filtrate. The peptide content was measured by UV light spectral density at 280 nm using an extinctions coefficient of 1.1 of 0.1% (g/L) solution that was calculated on the basis of the frequency of tryptophan and tyrosine in vertebrate proteins.

For labeling, each iTRAQ reagent in 70 μL ethanol was added to the respective peptide mixture. The samples were labeled as (Sample 1)-114, (Sample 2)-115, (Sample 3)-116 and (Sample 4)-117, and then multiplexed and vacuum dried. 

iTRAQ labeled peptides were fractionated by Strong Cation Exchange (SCX) chromatography using the AKTA Purifier system (GE Healthcare). The dried peptide mixture was reconstituted and acidified with 2 mL buffer A (10 mM KH_2_PO_4_ in 25% of ACN, pH 2.7), and then loaded into a 4.6 × 100 mm Polysulfoethyl column (PolyLC Inc., Maryland, MD, USA). Then, the peptides were eluted at a flow rate of 1 mL/min with a gradient of 0–10% buffer B (500 mM KCl, 10 mM KH_2_PO_4_ in 25% of ACN, pH 2.7) for 2 min, 10–20% buffer B for 25 min, 20–45% buffer B for 5 min and 50–100% buffer B for 5 min, respectively. Elution procedure was monitored by absorbance at 214 nm, and fractions were collected every 1 min. The collected fractions (about 30 fractions) were finally combined into 10 pools and desalted on C18 Cartridges (Empore™ SPE Cartridges C18 (standard density), bed I.D. 7 mm, volume 3 mL, Sigma). Each fraction was concentrated by vacuum centrifugation and then reconstituted in 40 µl of 0.1% (*v/v*) trifluoroacetic acid. All samples were stored at −80 °C and used for LC-MS/MS analysis.

Liquid Chromatography (LC)-Electrospray Ionization (ESI) Tandem MS (MS/MS) was analyzed by Q Exactive. Experiments were performed on a Q Exactive mass spectrometer that was coupled to Easy nLC (Proxeon Biosystems, now Thermo Fisher Scientific). Ten μL of each fraction was used for nano LC-MS/MS analysis. The peptide mixture (5 μg) was loaded onto a C18-reversed phase column (Thermo Scientific Easy Column, 10 cm long, 75 μm inner diameter, 3 μm resin) in buffer A (0.1% Formic acid) and separated with a linear gradient of buffer B (80% acetonitrile and 0.1% Formic acid) at a flow rate of 250 nl/min handled by IntelliFlow technology exceed 140 min. MS data were obtained using a data-dependent top 10 method, dynamically choosing the most abundant precursor ions from the survey scan (300–1800 *m*/*z*) for high energy collisional dissociation (HCD) fragmentation. Determination of the target value is based on predictive Automatic Gain Control (pAGC). Dynamic exclusion duration was 60 s. Survey scans were acquired at a resolution of 70,000 at *m*/*z* 200 and resolution for HCD spectra was set to 17,500 at *m*/*z* 200. Normalized collision energy was 30 eV and the underfill ratio, which specifies the minimum percentage of the target value likely to be reached at maximum fill time, was defined as 0.1%. The instrument was run with peptide recognition mode enabled.

The MS/MS spectra were searched against the uniport Sus scrofa database for peptide identification and quantification using Mascot 2.2 (Matrix Science, London, UK) and Proteome Discoverer1.3 (Thermo Electron, San Jose, CA, USA). The following options were used to identified protein: Peptide mass tolerance = 20 ppm, MS/MS tolerance = 0.1 Da, Enzyme = Trypsin, Missed cleavage = 2, Fixed modification: Carbamidomethyl (C), iTRAQ4/8plex(K), iTRAQ4/8plex(N-term), Variable modification: Oxidation(M), false discovery rate (FDR) ≤ 0.01. Additionally, fold change of ≥1.20 or ≤0.83, *t*-test *p*-value < 0.05 and FDR < 0.01 were set as the threshold to identify differentially expressed proteins (DEPs).

### 2.8. Statistical Analysis

The data of growth performance, carcass traits and sensory evaluation of finishing pigs were analyzed using SAS software (SAS 8.0, Inst, Inc., Cary, NC, USA), and presented as means ± SEM. Statistical significance was considered at *p* < 0.05. Growth performance was analyzed using the following model: y = μ + α + β, where y is the response variable, μ is the overall mean, α is the fixed effect of diet and β is the random effect of pen. Carcass traits and sensory evaluation analysis were analyzed using y = μ + α + β model, where y is the response variable, μ is the overall mean, α is the fixed effect of diet and β is the random effect of pig identification, panelist number and tasting sample order. The other traits were analyzed using GraphPad Prism Version 5 (GraphPad Software, La Jolla, CA, USA) and presented as means ± SEM. Significance was evaluated using a two-tailed unpaired *t* test. *p* < 0.05was considered to be a statistically significant difference.

## 3. Results

### 3.1. Characterization of Diets

As shown in Table 3, compared with control diet, fermented diet had lower dry matter, crude fiber and pH value. However, fermentation significantly increased the crude protein content and lactic acid concentration. Additionally, fermented diet was greater in aspartic acid, serine, glutamic acid, glycine, alanine, cysteine, valine, methionine, isoleucine, leucine, tyrosine, phenylalanine, lysine, histidine and proline when compared with the control diet.

### 3.2. Diets Effect on Growth Performance, Carcass Traits and Meat Quality of LT in Finishing Pigs 

As shown in Table 4, diets had no significant effect on ADG and ADFI, while the fermented diet tended to decrease F/G (*p* < 0.1). The fermented diet significantly decreased the last rib fat depth (*p* < 0.01) while increasing the loin eye area and lean mass percentage compared with the control diet (*p* < 0.01). The meat quality indices of LT are summarized in Table 5. The pH at 45 min, 24 h and 48 h, meat color scores (lightness, redness and yellowness values), drip loss at 24 h after slaughter and intramuscular fat content did not differ between pigs fed the fermented and the control diet. Meat from pigs fed the fermented diet had a significant decrease in drip loss at 48 h after slaughter (*p* < 0.05) and shear force (*p* < 0.01) compared to that from pigs fed the control diet. Additionally, the fermented diet significantly increased the scores for tenderness, fragrance and broth freshness (*p* < 0.05) but had no effect on the other sensory parameters including juiciness, flavor off-flavors and color of LT (Table 6). 

### 3.3. Diets Effect on Expression of Genes and Proteins Involved in Muscle Growth in LT 

**Figure 1:**
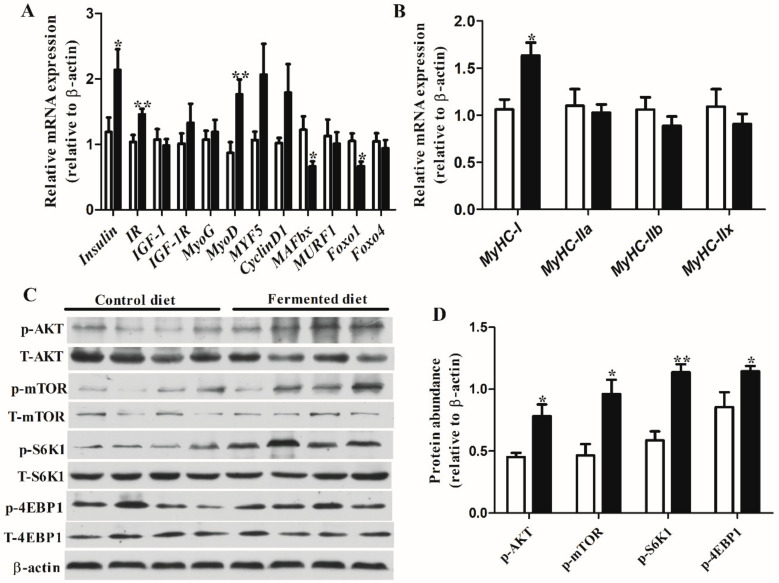
Diets effect on indices related to protein synthesis of finishing pigs. Relative abundance transcripts of genes (**A**) Insulin, *IR*, *IGF-1*, *IGF-1R*, *MyoD*, *MyoG*, *MYF5* and CyclinD1, *MAFbx*, *MURF1*, *FoxO1* and *FoxO4* in longissimus thoracis of finishing pigs. Relative abundance transcripts of genes (**B**) *MyHC-I, MyHC-IIa*, *MyHC-IIb* and *MyHC-IIx* in longissimus thoracis of finishing pigs. (**C**) Western blots illustrating the total and phosphorylation level of AKT, mTOR, S6K1 and 4EBP1 in longissimus thoracis. Equal protein loading was confirmed by analysis of β-actin in the protein extracts. (**D**) The ratios of p-AKT, p-mTOR, p-S6K1 and p-4EBP1 to β-actin. Data are means ± SEM. * *p* < 0.05, ** *p* < 0.01. *IR*, insulin receptor; *IGF-1*, insulin like growth factor 1; *IGF-1R*, insulin-like growth factor 1 receptor; *FoxO1*, forkhead Box O1; *FoxO4*, forkhead Box O4; *MyoD*, myoblast determination protein; *MyoG*, myogenin; *MYF5*, myogenic factor 5; *MAFbx*, muscle atrophy F-box; *MURF1*, Ring finger 1; *MyHC-I*, myosin heavy chain (MyHC)-I; *MyHC-IIa*, myosin heavy chain (MyHC)-IIa; *MyHC-IIb*, myosin heavy chain (MyHC)-IIb; MyHC-IIx, myosin heavy chain (MyHC)-IIx; mTOR, mammalian target of rapamycin; 4EBP1, initiation factor 4E-binding protein 1; S6K1, S6 kinase 1.

Figure 1A shows fermented diet significantly increased the expression of insulin (*p* < 0.05), *IR* (*p* < 0.01) and *MyoD* (*p* < 0.01), and the same trend for the expression of myogenic factor 5 (*MYF5*) and cyclinD1 mRNA, although there were no significant statistics, while decreasing the abundance of *MAFbx* and *Foxo1* transcripts (both *p* < 0.05). The fermented diet had no effect on the expression of insulin like growth factor 1 (*IGF-1*), insulin-like growth factor 1 receptor (*IGF-1R*), myogenin (*MyoG*), muscle ring finger 1(*MURF1*) and forkhead box O4 (*Foxo4*) compared with the control diet. As for the *MyHC* genes expression levels, the results showed that the fermented diet significantly increased the abundance of *MyHC-I*, and had no effect on fast-oxidative type IIa (*MyHC-IIa*), fast-oxidative glycolytic type IIx (*MyHC-IIx*) and fastglycolytic IIb (*MyHC-IIb*) (Figure 1B). At the protein level, western blots showed that the fermented diet markedly increased the phosphorylation of AKT (*p* < 0.05), mammalian target of rapamycin (mTOR) (*p* < 0.05), S6 kinase 1(s6k1) (*p* < 0.01) and initiation factor 4E-binding protein 1(4EBP1) (*p* < 0.05) compared with the control diet (Figure 1C,D).

### 3.4. Diets Effect on Proteomics Profiles of LT 

**Figure 2:**
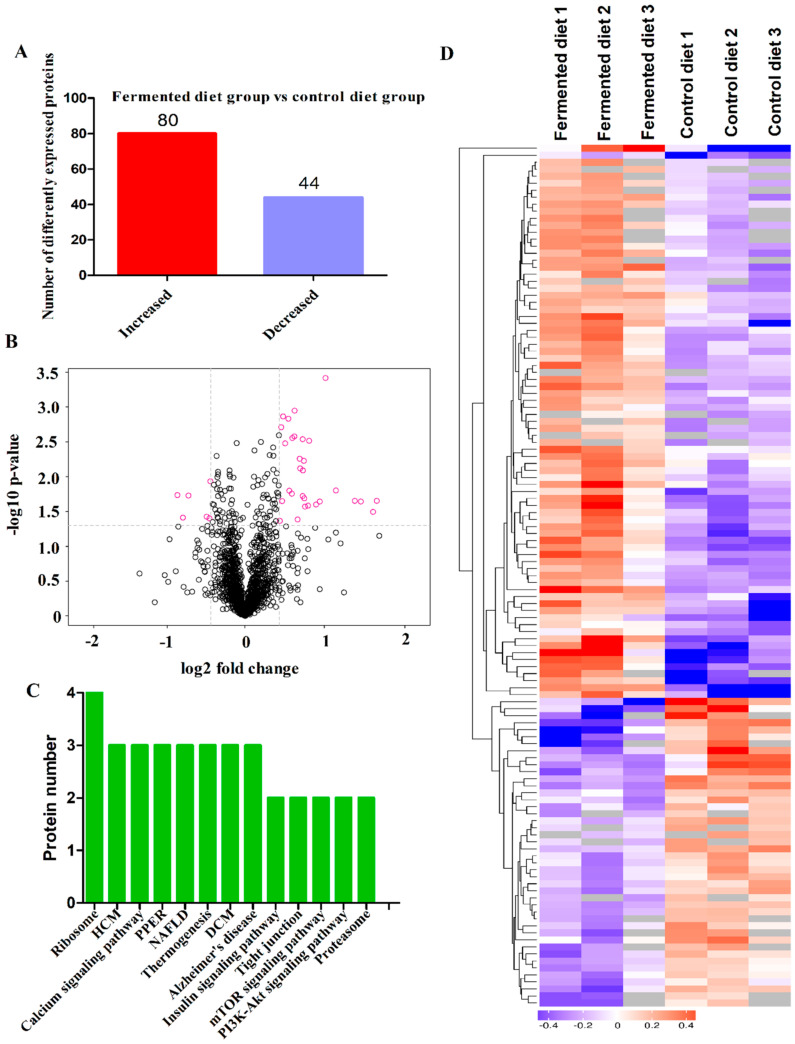
Statistical analysis of differentially expressed proteins in pigs fed the fermented diet compared with those fed the control diet. (**A**) The classification of differentially expressed proteins. (**B**) Volcano plots of identified proteins in pigs fed fermented diet compared with control diet; the red circles were the proteins that exhibited a differential expression pattern in pigs fed the fermented diet compared with those fed the control diet. Black represented no significant change in expression level. (**C**) Clustering analysis of the proteins differentially expressed between pigs fed the fermented diet and the control diet. Red and blue colored bars showed increased and decreased proteins, respectively. (**D**) The top 15 terms of Genes and Genomes (KEGG) pathway for differentially expressed proteins (DEPs) in pigs fed the fermented diet and the control diet. DCM, dilated cardiomyopathy; HCM, hypertrophic cardiomyopathy; NAFLD, non-alcoholic fatty liver disease; PPER, protein processing in endoplasmic reticulum.

**Figure 3:**
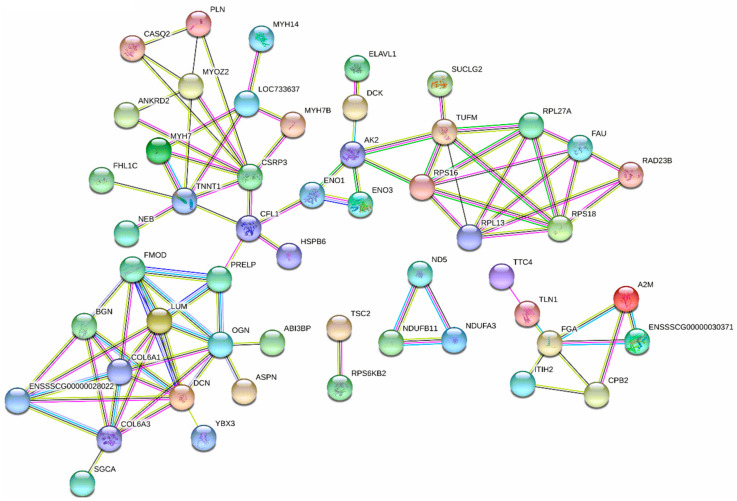
Interaction network of important DEPs (ribosomal proteins, SLRP-type proteoglycans and the collagen proteins and muscle contraction and muscle hypertrophy-related proteins) identified using iTRAQ in pigs fed the fermented diet compared with the control diet. ASPN, asporin; COLA1, collagen type VI alpha 2 chain; COL6A2, collagen type VI alpha 2 chain; COLA3, collagen type VI alpha 3 chain; CSRP3, cysteine and glycine-rich protein 3; DCN, decorin; DEPs, differentially expressed proteins; FMOD, fibromodulin; LUM, lumican; MYH7B, myosin heavy chain 7B; MYH7, myosin-7; OGN, osteoglycin; PRELP, proline and arginine rich end leucine rich repeat protein; RPS18, 40S ribosomal protein S18; RPL27A, 60S ribosomal protein L27a; RPL13, 60S ribosomal protein L13; FAU,40S ribosomal protein S30; RPS16, 40S ribosomal protein S16; TUFM, elongation factor Tu. Network nodes represent proteins; empty nodes 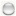: proteins of unknown three-dimensional structure; filled nodes 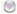: some three-dimensional structure is known or predicted; colored nodes: query proteins and first shell of interactors. Edges represent protein-protein associations; known Interactions: 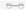 from curated databases and 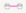 experimentally determined; predicted interactions: 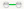 gene neighborhood, 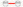 gene fusions and 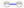 gene co-occurrence. Others: 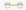 text mining, 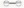 co-expression and 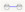 protein homolog.

The analysis identified 1549 proteins in LT, and 124 of them were DEPs between the two diets. Compared with the control diet, 80 DEPs were increased while 44 DEPs were decreased by the fermented diet (Figure 2A). VolcanoPlot analysis showed the expression changes of all proteins with quantitative iTRAQ ratios in Figure 2B and the red circles were the proteins that exhibited a differential expression pattern in pigs fed the fermented diet compared with those fed the control diet. Clusters analysis in different colors was used to show DEPs profiles between the two treatment groups, and all the DEPs are shown in Figure 2C. Kyoto Encyclopedia of Genes and Genomes (KEGG) analysis showed that significant enrichment of DEPs were predominantly associated with ribosome (four proteins), cardiac muscle contraction (three proteins), hypertrophic cardiomyopathy (three proteins), tight junction (two proteins), proteoglycans (two proteins), mTOR signaling pathway (two proteins) and PI3K-Akt signaling pathway (two proteins) (Figure 2D). The data of interaction networks of 124 DEPs showed that protein predominantly enriched in protein synthesis and muscle development (Figure 3). 40S ribosomal protein S18 (RPS18), 60S ribosomal protein L27a (RPL27A), 60S ribosomal protein L13 (RPL13), 40S ribosomal protein S30 (FAU), 40S ribosomal protein S16 (RPS16) and elongation factor Tu (TUFM), which contributed to protein synthesis, played a pivotal role in the protein interaction network. Of these, DEPs, FAU, RPS16, RPS18, RPL27A and RPL13 were increased by the fermented diet, but TUFM was decreased (Table 7). Regulatory extracellular matrix complexome (ECM) proteins including asporin (ASPN), lumican (LUM), proline and arginine rich end leucine rich repeat protein (PRELP), osteoglycin (OGN) proteins, Decorin (DCN), Fibromodulin (FMOD), Biglycan (BGN) and collagen type VI alpha 1 chain (COL6A1), collagen type VI alpha 2 chain (COL6A2) and collagen type VI alpha 3 chain (COL6A3) were significantly increased by the fermented diet (Table 7). Additionally, myosin heavy chain 7B (MYH 7B), myosin-7 (MYH7), troponin T (TNNT1) and cysteine and glycine-rich protein 3 (CSRP3) played an important role in the network. The fermented diet increased the proteins expression of MYH7B, MYH7 and CSRP3, but reduced the protein level of TNNT1 (Table 7).

### 3.5. Correlation Analysis between Percentage Lean Meat, Gene Expression and Proteins Related to Muscle Growth

**Figure 4:**
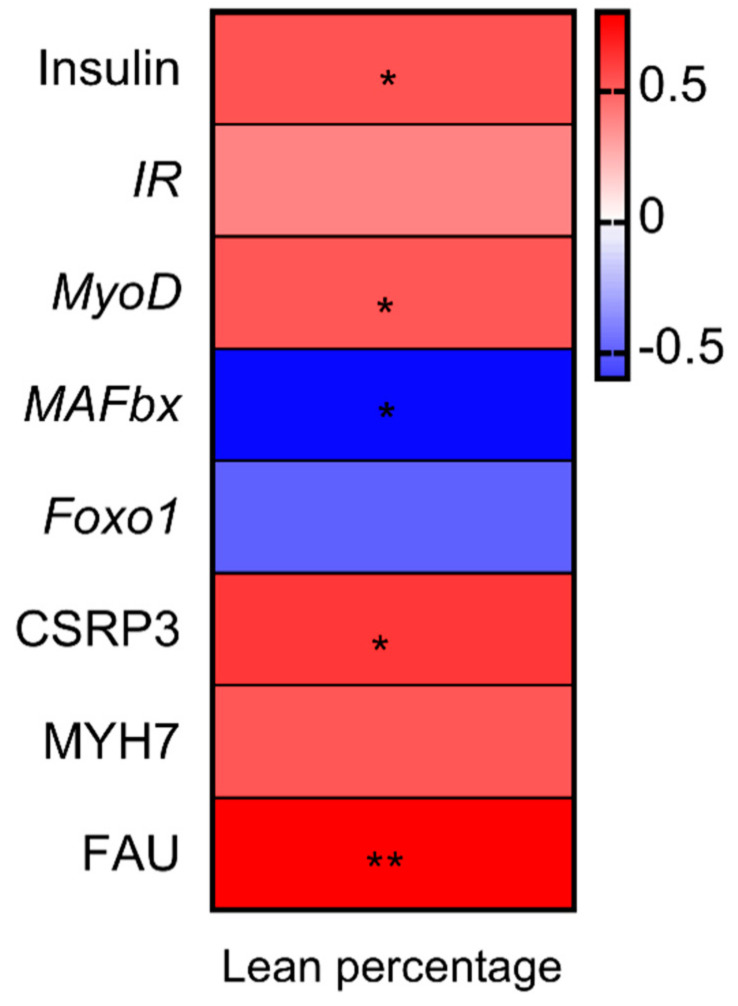
Heatmap of Pearson’s rank correlation coefficients between the relative abundance of genera, ADG, bacterial metabolites and intestinal genes expression. In the panels, * *p* < 0.05 and ** *p* < 0.01. *IR*, insulin receptor; *MyoD*, myoblast determination protein; MAFbx, muscle atrophy F-box; Foxo1, forkhead Box O1; FAU, 40S ribosomal protein S30; MYH7, myosin-7; CSRP3, cysteine and glycine-rich protein 3.

A Pearson correlation analysis was used to investigate the associations between genes and proteins expression and lean percentage (Figure 4). The results revealed that lean percentage had significantly positive correlation with the expression insulin, *MyoD*, CSRP3 and FAU, while it was negatively related to the abundance of *MAFbx*.

## 4. Discussion

In the present study, we found that microbial fermentation decreased crude fiber and pH value, and increased the lactic acid concentration, crude protein content and amino acid profiles of the feed. The increased crude protein after fermentation was consistent with previous fermented soybean meal (SBM) [8,25], fermented rapeseed meal [26] and fermented cottonseed meal [27]. The increase in protein may be attributed to bacterial growth and proliferation, as well as mycelia and/or enzymes produced by probiotics [28,29]. Additionally, the loss of dry matter during fermentation could be another possible reason for an increase in crude protein [30]. Consistent with previous studies, fermentation was able to increase aspartic acid, glutamic acid, serine, alanine, proline, valine, methionine, cysteine, isoleucine, phenylalanine, tyrosine, leucine, histidine and lysine levels. Improvement in the crude protein content and amino acid profile of the feed in response to fermentation may showed a beneficial effect on growth performance and meat quality of finishing pigs.

Several studies have reported that fermented feed had no significant effect on ADFI and ADG of finishing pigs [16,17]. Consistent with these results, the results in the present study showed that there was no significant effect of fermented diet on growth performance of finishing pigs. However, fermented feed has been shown to improve meat quality of finishing pigs. Study by Fang et al. reported that feeding fermented apple pomace to finishing pigs reduced the drip loss [31]. Additionally, a study by Kim et al. reported dietary supplementation of fermented persimmon shell improved sensory tenderness in pork longissimus muscle [18]. Consistent with these results, the present study showed that fermented feed effectively improved meat quality by reducing the drip loss (48 h) and shear force. Additionally, a fermented diet enhanced meat tenderness, fragrance and broth freshness. These sensory characteristics have been regarded as critical markers for eating quality of meat because they influence repeat purchases by consumers [32]. The increase in the abundance of *MyHC-I*, which contributes to increase water holding capacity and tenderness [33], further confirmed that fermented diet in the present study could improve meat quality of finishing pig without affecting intramuscular fat (IMF)I

In the present study, the fermented diet significantly increased loin eye area and lean mass percentage. A study by Ahmed et al. showed that fermented herb diet increased lean production [20], but the mechanisms underlying this observation remain unknown. Increased amino acid profile of the feed after fermentation may play an important role in skeletal muscle accretion through increased protein synthesis signaling and suppressed degradation signaling in skeletal muscle of LT in finishing pigs [34,35]. Insulin and its receptor play important roles in protein synthesis signaling pathways [36]. Binding of insulin to its receptor triggers activation of the phosphatidylinositol 3-kinase (PI3K)-Akt pathway and subsequent activation of mTOR; activation of mTOR results in the phosphorylation and activation of S6K1 and 4EBP1, which in turn phosphorylates ribosomal protein S6 and, finally, stimulates protein synthesis [37,38]. Previous studies have indicated that amino acids, especially leucine play an important role promoting muscle protein synthesis via stimulating mammalian target of rapamycin (mTOR) pathway activity in skeletal muscle of piglets and cells models [39,40,41]. In the present study, fermented diet increased the expression of insulin and its receptor, and activated the phosphorylation of AKT, mTOR, S6K1 and 4EBP1. Therefore, the increased lean production in response to the increased amino acid profile after fermentation may be due to, at least in part, the activation of insulin/AKT/mTOR signaling pathway in the skeletal muscle of LT. 

The modulation of skeletal muscle mass depends on the balance between protein synthesis and degradation. Previous studies have shown a reduction in protein degradation after branched-chain amino acids or leucine administration through changing the expression of Foxo1, MAFbx and MuRF-1 [42,43,44,45], which play an essential role in promoting muscle proteolysis [46]. Consistent with these studies, our result showed that increased amino acid profile after fermentation decreased the abundance of *Foxo1* and *MAFbx*. Additionally, a previous study reported that leucine also increased the mRNA and protein levels of MyoG and MyoD [42], which was an important regulator of myogenesis [47]. Similarly, we also observed an increased expression of *MyoD* by the fermented feed that was rich in amino acid profile. Consequently, the proteolysis-related FOXO1/MAFbx pathway and protein synthesis-related MyoD pathway may serve as a target for muscle metabolism and growth when subjected to increased amino acid profile of the feed after fermentation.

On the other hand, ribosomal proteins and muscle contraction-related proteins have been shown to play an important role in protein synthesis during myogenesis and promote muscle growth [48,49,50,51]. In this study, protein interaction network prediction using iTRAQ revealed that proteins from 60 S ribosomal subunit (RPL27A and RPL13) and 40 S ribosomal subunit (FAU, RPS18 and RPS16), as well as proteins (MYH7B, MYH7, CSRP3) involved in muscle contraction and muscle hypertrophy were increased by fermented diet. In addition, the increase in the expression of muscle-associated proteoglycans of the type SLRP including ASPN, LUM, PRELP, DCN, FMOD, BGN and OGN, and collagen isoform including COL6A1, COL6A2 and COL6A3 indicated that fermented feed regulated the development and growth of skeletal muscles [52,53]. Therefore, these results can be an additional support for regulation of protein metabolism in skeletal muscle of LT by increased amino acid profile of the feed after fermentation, which may promote muscle deposition.

## 5. Conclusions

In summary, the promotion of muscle growth by the increased amino acid profile of the feed after fermentation was regulated, at least in part, by increasing the insulin/AKT/mTORC1 protein synthesis cascade and activating Foxo1/MAFbx pathway, along with the regulation of ribosomal protein and proteins involved in muscle contraction and muscle hypertrophy. Additionally, a fermented diet improved meat quality of longissimus thoracis (LT) by decreasing the shear force and drip loss at 48 h post slaughter and increasing sensory characteristics without affecting the IMF content. 

## Figures and Tables

**Table 1:** Composition of the basal diet.

Ingredients, %	%	Nutrient Content ^2^	
yellow corn (8.1% CP)	66	Digestible energy, kcal/kg	3386.63
soybean meal (46% CP)	13	Metabolizable energy, kcal/kg	3233.67
soybean hulls	15	Net energy, kcal/kg	2433.02
L-Lys-HCL	0.2	Crude protein	13.03
L-Thr	0.05	Calcium	0.68
soybean oil	2	Total phosphorus	0.50
limestone powder	0.8	Available phosphorus	0.28
Calcium monohydrogen phosphate	1	Standardized ileal digestible Lys	0.66
sodium chloride	0.95	Standardized ileal digestible Met	0.19
Premix ^1^	1	Standardized ileal digestible Met + cys	0.4
Total	100	Standardized ileal digestible Thr	0.44
		Standardized ileal digestible Trp	0.11

^1^ Supplied per kilogram of the complete diet: Fe, 120 mg; Cu, 10 mg; Zn, 120 mg; Mn, 35 mg; I, 0.25 mg, Se, 0.2 mg; vitamin A, 8000 IU; vitamin D3, 1000 IU; vitamin E, 30 mg; vitamin K3, 2 mg; vitamin B1, 2 mg; vitamin B2, 6 mg; vitamin B6, 4.0 mg; vitamin B12, 0.02 mg; niacin, 25 mg; calcium pantothenate, 10 mg; folic acid, 1.0 mg; biotin, 0.25 mg. ^2^ All values were calculated based on database of NRC (2012).

**Table 2:** Primer sequences used in this study.

Genes	Sequences (5′–3′)		Product Size (bp)	GenBank Accession
*MAFbX*	Forward	CCCTCTCATTCTGTCACCTTG	104	NM_001044588
	Reverse	ATGTGCTCTCCCACCATAGC		
*MyoG*	Forward	CTTCTACCAGGAACCCCACT	230	NM_001012406
	Reverse	TCCCCAGCCCCTTATCTT		
*MyoD*	Forward	ATGATGACCCGTGTTTCG	383	NM_001002824
	Reverse	GCCTCGTTGACTTTGCTC		
*MuRF1*	Forward	GCTGGATTGGAAGAAGATGTAT	144	NM_001184756
	Reverse	AGGAAAGAATGTGGCAGTGTCT		
*IGF-1*	Forward	TCTTCAGTTCGTGTGCGGAG	165	NM_214256
	Reverse	TTGGCAGGCTTGAGGGGT		
*IGF-1R*	Forward	ATGGAGGAAGTGACAGGGACTA	116	XM_003361272
	Reverse	GTGGTGGTGGAGGTGAAGTG		
*Foxo1*	Forward	F: CGGCATCATCTTCATCGTC 125	125	NM_214014.2
	Reverse	R: CTGTCCTCCCACTCCAGGTA		
*Foxo4*	Forward	F: CTGTCCTACGCCGACCTCAT 103	103	NM_003135172.2
	Reverse	R: TTGCTGTCACCCTTATCCTTG		
*IR*	Forward	F: CATACCTGAACGCCAAGAAGTT	100	XM_003123154
	Reverse	R: GTCATTCCAAAGTCTCCGATTT		
Insulin	Forward	CGCGGCTTCTTCTACAC	134	NM_001109772
	Reverse	ACGATGCCACGCTTCTG		
*CyclinD1*	Forward	AGGTTGGGAGTGCGTTG	166	XM_021082686.1
	Reverse	TTGGCGGAGATTTGGAG		
*MYF5*	Forward	GTTCGGGGACGAGTTTG	272	NM_001278775.1
	Reverse	GCCTCTGGTTGGGGTTA		
*MyHC-I*	Forward	GGCCCCTTCCAGCTTGA	63	L10129
	Reverse	TGGCTGCGCCTTGGTTT		
*MyHC-IIa*	Forward	TTAAAAAGCTCCAAGAACTGTTTCA	100	U11772
	Reverse	CCATTTCCTGGTCGGAACTC		
*MyHC-IIb*	Forward	CACTTTAAGTAGTTGTCTGCCTTGAG	80	U90720
	Reverse	GGCAGCAGGGCACTAGATGT		
*MyHC-IIx*	Forward	AGCTTCAAGTTCTGCCCCACT	76	U90719
	Reverse	GGCTGCGGGTTATTGATGG		
*β-actin*	Forward	CATCGTCCACCGCAAAT	210	NC_010445
	Reverse	TGTCACCTTCACCGTTCC		

**Table 3:** Chemical composition and biochemical indexes of experimental diets.

Item	Control Diet	Fermented Diet	*p* Value
Analyzed Chemical Composition ^1^			
Dry matter (%)	87.95 ± 0.10	62.19 ± 0.29	<0.01
Crude fiber (%)	6.72 ± 0.36	6.29 ± 0.22	0.04
Neutral detergent fiber (%)	15.05 ± 1.01	15.85 ± 0.53	0.85
Acid detergent fiber (%)	7.22 ± 0.32	8.64 ± 0.18	0.44
Crude protein (%)	12.94 ± 0.07	13.77 ± 0.09	<0.01
Crude fat (%)	4.15 ± 0.06	4.55 ± 0.20	0.43
Profile of Amino Acids (mg 100 g^−1^)			
Aspartic acid	1.17 ± 0.01	1.27 ± 0.02	<0.01
Threonine	0.52 ± 0.00	0.53 ± 0.01	0.43
Serine	0.62 ± 0.01	0.66 ± 0.01	0.01
Glutamic acid	2.33 ± 0.03	2.40 ± 0.03	0.02
Glycine	0.51 ± 0.01	0.66 ± 0.01	<0.01
Alanine	0.73 ± 0.01	0.83 ± 0.01	<0.01
Cysteine	0.16 ± 0.00	0.22 ± 0.01	<0.01
Valine	0.61 ± 0.01	0.67 ± 0.01	<0.01
Methionine	0.09 ± 0.00	0.09 ± 0.00	0.66
Isoleucine	0.52 ± 0.01	0.58 ± 0.01	0.00
Leucine	1.18 ± 0.00	1.30 ± 0.01	<0.01
Tyrosine	0.22 ± 0.01	0.35 ± 0.01	<0.01
Phenylalanine	0.61 ± 0.01	0.66 ± 0.01	0.01
Lysine	0.87 ± 0.01	0.92 ± 0.01	0.02
Histidine	0.35 ± 0.00	0.38 ± 0.01	0.01
Arginine	0.67 ± 0.01	0.65 ± 0.01	0.25
Proline	0.82 ± 0.01	0.90 ± 0.01	<0.01
Other Biochemical Indexes			
pH	6.11 ± 0.00	4.88 ± 0.03	<0.01
Lactic acid (mmol/kg)	23.56 ± 1.34	165.07 ± 5.25	<0.01

Values are means ± SEM, *n* = 3/treatment. ^1^ Analytical results obtained according to AOAC (AOAC, 2007). Values are means ± SEM, statistical significance was considered at *p* < 0.05. Crude fiber, neutral detergent fiber, acid detergent fiber, crude fat, starch, crude protein contents and amino acids were measured on an 88% dry matter basis.

**Table 4:** Diets effect on the growth performance and carcass traits in finishing pigs.

		Control Diet	Fermented Diet	*p* Value
Initial BW (kg)		98.86 ± 0.32	98.25 ± 0.27	0.16
Final BW (kg)		130.78 ± 1.14	134.93 ± 1.97	0.66
ADG (kg)		0.84 ± 0.03	0.94 ± 0.10	0.27
ADFI (kg, on an 88% DM basis)		3.34 ± 0.12	3.06 ± 0.11	0.13
F/G		4.05 ± 0.15	3.29 ± 0.08	0.07
Loin eye area (cm^2^)		61.56 ± 2.47	70.61 ± 2.25	0.03
Lean mass (%)		53.1 ± 1.26	61.3 ± 1.72	<0.01
Backfat depth (cm)	First rib	4.36 ± 0.34	4.49± 0.31	0.67
	10th rib	3.13 ± 0.34	3.01 ± 0.23	0.80
	Last rib	2.67 ± 0.15	2.11 ± 0.16	0.03

Values are means ± SEM, statistical significance was considered at *p* < 0.05. ADG: average daily gain; ADFI: average daily feed intake; F/G: feed to gain ratio; DM: dry matter, BW: body weight.

**Table 5:** Diets effect on the meat quality of longissimus thoracis muscle.

	Control Diet	Fermented Diet	*p* Value
Drip loss_24h_ (%)	2.55 ± 0.15	2.43 ± 0.07	0.46
Drip loss_48h_ (%)	3.85 ± 0.14	3.08 ± 0.23	0.01
Shear force (N)	66.78 ± 4.50	49.96 ± 3.10	0.01
pH_45min_	5.94 ± 0.11	6.07 ± 0.06	0.36
pH_24h_	5.51 ± 0.03	5.53 ± 0.03	0.92
pH_48h_	5.61 ± 0.03	5.51 ± 0.03	0.09
L * (lightness)_45min_	46.63 ± 0.94	46.14 ± 0.62	0.66
a * (redness)_45min_	16.82 ± 0.60	16.66 ± 0.62	0.81
b * (yellowness)_45min_	1.71 ± 0.38	2.01 ± 0.18	0.47
L * (lightness)_24h_	54.93 ± 0.54	54.47 ± 0.15	0.77
a * (redness)_24h_	17.67 ± 0.48	17.18 ± 0.28	0.40
b * (yellowness)_24h_	2.21 ± 0.25	2.46 ± 0.25	0.48
L * (lightness)_48h_	54.60 ± 0.82	54.33 ± 0.15	0.85
a * (redness)_48h_	18.72 ± 0.59	17.90 ± 0.49	0.31
b * (yellowness)_48h_	1.82 ± 0.31	2.41 ± 0.26	0.18
Intramuscular fat (%)	1.22 ± 0.11	1.13 ± 0.26	0.75

Values are means ± SEM (*n* = 8), statistical significance was considered at *p* < 0.05. * CIELab.

**Table 6:** Sensory traits evaluation of grilled longissimus thoracis muscle.

	Control Diet	Fermented Diet	*p* Value
Cooked Pork Sensory Traits			
Juiciness	4.52 ± 0.10	4.48 ± 0.13	0.56
Tenderness	4.26 ± 0.22	4.73 ± 0.11	0.04
Flavor	4.72 ± 0.07	4.96 ± 0.10	0.07
Fragrance	4.79 ± 0.07	5.18 ± 0.10	0.02
Off-flavors	5.13 ± 0.07	5.27 ± 0.17	0.29
Broth freshness	4.85 ± 0.07	5.31 ± 0.10	0.01
Color	4.89 ± 0.09	5.15 ± 0.1	0.10

Values are means ± SEM (*n* = 8), statistical significance was considered at *p* < 0.05. Sensory variables were rated using 8-point scales: tenderness (1 = very tough, 8 = very tender), juiciness (1 = very dry, 8 = very juicy), flavor (1 = very poor, 8 = very good), fragrance (1 = very weak, 8 = very strong), off-flavors (1 = very strong, 8 = very weak), broth freshness (1 = very poor, 8 = very strong) and color (1 = pale, 8 = dark purplish red).

**Table 7:** Differentially expressed proteins related to muscle growth in pigs.

Protein IDs	Gene Name	Protein Name	Fold Change	*p* Value
Protein Synthesis				
F1RFI1	TUFM	Elongation factor Tu	0.82	0.04
P62272	RPS18	40S ribosomal protein S18	1.29	0.01
K7GKC0	RPS16	40S ribosomal protein S16	1.83	0.05
A0A287AWS4	RPL27A	60S ribosomal protein L27a	1.30	0.03
I3LSD3	RPL13	60S ribosomal protein L13	1.33	0.02
P62863	FAU	40S ribosomal protein S30	1.56	0.001
SLRP-type proteoglycans and collagens				
F1SUE4	ASPN	Aspirin	1.24	0.03
F1SQ09	LUM	Lumican	1.31	0.04
A0A0H5ANC0	OGN	Osteoglycin	1.45	0.00
F1S6B4	PRELP	Proline and arginine rich end leucine rich repeat protein	1.73	0.01
F1S6B5	FMOD	Fibromodulin	2.24	0.05
F1S2B6	BGN	Biglycan	1.70	0.02
Q9XSD9	DCN	Decorin	1.51	0.01
Q1T7A8	COL6A1	Type VI collagen alpha-1 chain	1.47	0.01
I3LQ84	COL6A2	Collagen type VI alpha 2 chain	1.48	0.02
A0A287BPF4	COL6A3	Collagen type VI alpha 3 chain	1.65	0.00
Muscle contraction and hypertrophy				
F1S4 × 7	MYH7B	Myosin heavy chain 7B	1.32	0.01
P79293	MYH7	Myosin-7	1.68	0.03
Q75ZZ6	TNNT1	Troponin T	0.68	0.02
F6Q6A7	CSRP3	Cysteine and glycine-rich protein 3	1.26	0.04

Fold change = Fermented diet/Control diet.

## References

[1] Plumed-Ferrer C., Von Wright A. (2009). Fermented pig liquid feed: nutritional, safety and regulatory aspects. J. Appl. Microbiol..

[2] Olstorpe M., Axelsson L., Schnurer J., Passoth V., Schnürer J. (2010). Effect of starter culture inoculation on feed hygiene and microbial population development in fermented pig feed composed of a cereal grain mix with wet wheat distillers’ grain. J. Appl. Microbiol..

[3] Kiarie E., Bhandari S., Scott M., Krause D.O., Nyachoti C.M. (2011). Growth performance and gastrointestinal microbial ecology responses of piglets receiving Saccharomyces cerevisiae fermentation products after an oral challenge with Escherichia coli (K88)1. J. Anim. Sci..

[4] Wang C., Shi C., Zhang Y., Song D., Lu Z., Wang Y. (2018). Microbiota in fermented feed and swine gut. Appl. Microbiol. Biotechnol..

[5] Zheng L., Li D., Li Z.-L., Kang L.-N., Jiang Y.-Y., Liu X.-Y., Chi Y.-P., Li Y.-Q., Wang J.-H. (2017). Effects of Bacillus fermentation on the protein microstructure and anti-nutritional factors of soybean meal. Lett. Appl. Microbiol..

[6] Shi C., He J., Yu J., Yu B., Mao X., Zheng P., Huang Z., Chen D. (2016). Physicochemical Properties Analysis and Secretome of Aspergillus niger in Fermented Rapeseed Meal. PLoS ONE.

[7] Le M.H.A., Galle S., Yang Y., Landero J.L., Beltranena E., Gänzle M.G., Zijlstra R.T. (2016). Effects of feeding fermented wheat with Lactobacillus reuteri on gut morphology, intestinal fermentation, nutrient digestibility, and growth performance in weaned pigs1. J. Anim. Sci..

[8] Shi C., Zhang Y., Yin Y., Wang C., Lu Z., Wang F., Feng J., Wang Y. (2017). Amino acid and phosphorus digestibility of fermented corn-soybean meal mixed feed with Bacillus subtilis and Enterococcus faecium fed to pigs1. J. Anim. Sci..

[9] Shi C., Zhang Y., Lu Z., Wang Y. (2017). Solid-state fermentation of corn-soybean meal mixed feed with Bacillus subtilis and Enterococcus faecium for degrading antinutritional factors and enhancing nutritional value. J. Anim. Sci. Biotechnol..

[10] Canibe N., Jensen B.B. (2003). Fermented and nonfermented liquid feed to growing pigs: effect on aspects of gastrointestinal ecology and growth performance. J. Anim. Sci..

[11] Wang N.F., Chen Q., Le G.W., Shi Y.H., Sun J. (2007). Effect of lactic acid fermented soybean meal on the growth performance, intestinal microflora and morphology of weaned piglets. J. Anim. Feed Sci..

[12] Missotten J., Michiels J., DeGroote J., De Smet S. (2015). Fermented liquid feed for pigs: an ancient technique for the future. J. Anim. Sci. Biotechnol..

[13] Yin F., Farzan A., Wang Q., Yu H., Yin J., Hou Y., Friendship R., Gong J. (2014). Reduction of salmonella enterica serovar typhimurium DT104 infection in experimentally challenged weaned pigs fed a lactobacillus-fermented feed. Foodborne Pathog. Dis..

[14] Lee S.D., Kim H.Y., Jung H.J., Ji S.Y., Chowdappa R., Ha J.H., Song Y.M., Park J.C., Kil Moon H., Kim I.C. (2009). The effect of fermented apple diet supplementation on the growth performance and meat quality in finishing pigs. Anim. Sci. J..

[15] Xu X., Li L.-M., Li B., Guo W.-J., Ding X.-L., Xu F.-Z. (2017). Effect of fermented biogas residue on growth performance, serum biochemical parameters, and meat quality in pigs. Asian-Australas. J. Anim. Sci..

[16] Liu P., Zhao J., Guo P., Lu W., Geng Z., Levesque C.L., Johnston L.J., Wang C., Liu L., Zhang J. (2017). Dietary corn bran fermented by bacillus subtilis MA139 decreased gut cellulolytic bacteria and microbiota diversity in finishing pigs. Front. Microbiol..

[17] Hu J., Lu W., Wang C., Zhu R., Qiao J. (2008). Characteristics of solid-state fermented feed and its effects on performance and nutrient digestibility in growing-finishing pigs. Asian-Australas. J. Anim. Sci..

[18] Kim H.-Y., Song Y.-M., Jin S.-K., Kim I.-S., Kang Y.-S., Lee S.-D., Chowdappa R., Ha J.-H., Kang S.-M. (2005). The effect of change in meat quality parameters on pig longissimus dorsi muscle by the addition of fermented persimmon shell diet. Asian-Australas. J. Anim. Sci..

[19] Yan L., Meng Q.W., Kim I.H. (2011). Effects of fermented garlic powder supplementation on growth performance, nutrient digestibility, blood characteristics and meat quality in growing-finishing pigs. Anim. Sci. J..

[20] Ahmed S.T., Mun H.-S., Islam M., Ko S.-Y., Yang C.-J. (2016). Effects of dietary natural and fermented herb combination on growth performance, carcass traits and meat quality in grower-finisher pigs. Meat Sci..

[21] Åby B.A., Kantanen J., Aass L., Meuwissen T. (2014). Current status of livestock production in the Nordic countries and future challenges with a changing climate and human population growth. Acta Agric. Scand. Sect. A Anim. Sci..

[22] Pandey A. (2003). Solid-state fermentation. Biochem. Eng. J..

[23] Luque de Castro M.D., Priego-Capote F. (2010). Soxhlet extraction: Past and present panacea. J. Chromatogr. A.

[24] Wiśniewski J.R., Zougman A., Nagaraj N., Mann M. (2009). Universal sample preparation method for proteome analysis. Nat. Methods.

[25] Chen C.C., Shih Y.C., Chiou P.W.S., Yu B. (2010). Evaluating nutritional quality of single stage- and two stage-fermented soybean meal. Asian-Australas. J. Anim. Sci..

[26] Shi C., He J., Yu J., Yu B., Mao X., Zheng P., Huang Z., Chen D. (2015). Amino acid, phosphorus, and energy digestibility of Aspergillus niger fermented rapeseed meal fed to growing pigs1. J. Anim. Sci..

[27] Sun H., Tang J.-W., Yao X.-H., Wu Y., Wang X., Liu Y., Lou B. (2015). Partial substitution of fish meal with fermented cottonseed meal in juvenile black sea bream (Acanthopagrus schlegelii) diets. Aquaculture.

[28] Raimbault M. (1998). General and microbiological aspects of solid substrate fermentation. Electron. J. Biotechnol..

[29] Lohlum S.A., Forcados E.G., Chuku A., Agida O.G., Ozele N. (2014). Corn cob as a feed component through fungal fermentation using Aspergillus niger. CIBTech. J. Microbiol..

[30] Rozan P., Villaum C., Bau H.M., Schwertz A., Nicolas J.P., Mejean L. (1996). Detoxication of rapeseed meal by Rhizopus Oligosporus sp-T3: A first step towards rapeseed protein concentrate. Int. J. Food Sci. Technol..

[31] Fang J., Cao Y., Matsuzaki M., Suzuki H., Kimura H. (2016). Effects of apple pomace-mixed silage on growth performance and meat quality in finishing pigs. Anim. Sci. J..

[32] Maltin C.A., Warkup C.C., Matthews K.R., Grant C.M., Porter A.D., Delday M.I. (1997). Pig muscle fiber characteristics as a source of variation in eating quality. Meat Sci..

[33] Ryu Y., Kim B. (2005). The relationship between muscle fiber characteristics, postmortem metabolic rate, and meat quality of pig longissimus dorsi muscle. Meat Sci..

[34] Fan Q., Long B., Yan G., Wang Z., Shi M., Bao X., Hu J., Li X., Chen C., Zheng Z. (2017). Dietary leucine supplementation alters energy metabolism and induces slow-to-fast transitions in longissimus dorsi muscle of weanling piglets. Br. J. Nutr..

[35] Zheng L., Wei H.-K., Cheng C., Xiang Q., Pang J., Peng J. (2016). Supplementation of branched-chain amino acids to a reduced-protein diet improves growth performance in piglets: involvement of increased feed intake and direct muscle growth-promoting effect. Br. J. Nutr..

[36] Saltiel A., Kahn C.R. (2001). Insulin signalling and the regulation of glucose and lipid metabolism. Nature.

[37] Manning B.D., Cantley L.C. (2007). AKT/PKB signaling: Navigating downstream. Cell.

[38] Suryawan A., Torrazza R.M., Gazzaneo M.C., Orellana R.A., Fiorotto M.L., El-Kadi S.W., Srivastava N., Nguyen H.V., Davis T.A. (2012). Enteral leucine supplementation increases protein synthesis in skeletal and cardiac muscles and visceral tissues of neonatal pigs through mTORC1-dependent pathways. Pediatr. Res..

[39] Zheng L., Wei H.-K., He P., Zhao S., Xiang Q., Pang J., Peng J. (2016). Effects of supplementation of branched-chain amino acids to reduced-protein diet on skeletal muscle protein synthesis and degradation in the fed and fasted states in a piglet model. Nutrients.

[40] Zhang B., Lin M., Yu C., Li J., Zhang L., Zhou P., Yang W., Gao F., Zhou G. (2016). Alanyl-glutamine supplementation regulates mTOR and ubiquitin proteasome proteolysis signaling pathways in piglets. Nutrients.

[41] Zhang S.-H., Ren M., Zeng X., He P., Ma X., Qiao S. (2014). Leucine stimulates ASCT2 amino acid transporter expression in porcine jejunal epithelial cell line (IPEC-J2) through PI3K/Akt/mTOR and ERK signaling pathways. Amino Acids.

[42] Zhang S.R., Chen X.L., Huang Z.Q., Chen D.W., Yu B., Chen H., Luo J.Q., He J., Zheng P., Yu J. (2018). Leucine promotes differentiation of porcine myoblasts through the protein kinase B (Akt)/Forkhead box O1 signalling pathway. Br. J. Nutr..

[43] Nakashima K., Ishida A., Yamazaki M., Abé H. (2005). Leucine suppresses myofibrillar proteolysis by down-regulating ubiquitin–proteasome pathway in chick skeletal muscles. Biochem. Biophys. Res. Commun..

[44] Louard R.J., Barrett E.J., Gelfand R.A. (1995). Overnight branched-chain amino acid infusion causes sustained suppression of muscle proteolysis. Metabolism.

[45] Maki T., Yamamoto D., Nakanishi S., Iida K., Iguchi G., Takahashi Y., Kaji H., Chihara K., Okimura Y. (2012). Branched-chain amino acids reduce hindlimb suspension-induced muscle atrophy and protein levels of atrogin-1 and MuRF1 in rats. Nutr. Res..

[46] Kousteni S. (2012). FoxO1, the transcriptional chief of staff of energy metabolism. Bone.

[47] Shi X., Garry D.J. (2006). Muscle stem cells in development, regeneration, and disease. Genes Dev..

[48] Lindström M.S. (2009). Emerging functions of ribosomal proteins in gene-specific transcription and translation. Biochem. Biophys. Res. Commun..

[49] De Klerk E., Fokkema I.F., Thiadens K.A., Goeman J.J., Palmblad M., Dunnen J.T.D., Von Lindern M., Hoen P.A.C. (2015). Assessing the translational landscape of myogenic differentiation by ribosome profiling. Nucleic Acids Res..

[50] Geier C., Gehmlich K., Ehler E., Hassfeld S., Perrot A., Hayess K., Cardim N., Wenzel K., Erdmann B., Krackhardt F. (2008). Beyond the sarcomere: CSRP3 mutations cause hypertrophic cardiomyopathy. Hum. Mol. Genet..

[51] Bian Z.-Y., Huang H., Jiang H., Shen D.-F., Yan L., Zhu L.-H., Wang L., Cao F., Liu C., Tang Q.-Z. (2010). LIM and cysteine-rich domains 1 regulates cardiac hypertrophy by targeting calcineurin/nuclear factor of activated T cells signaling. Hypertension.

[52] Goody M.F., Sher R.B., Henry C.A. (2015). Hanging on for the ride: adhesion to the extracellular matrix mediates cellular responses in skeletal muscle morphogenesis and disease. Dev. Biol..

[53] Ohlendieck K. (2016). The extracellular matrix complexome from skeletal muscle. Composition and Function of the Extracellular Matrix in the Human Body.

